# Moderate-density parity-check codes from projective bundles

**DOI:** 10.1007/s10623-022-01054-y

**Published:** 2022-05-24

**Authors:** Jessica Bariffi, Sam Mattheus, Alessandro Neri, Joachim Rosenthal

**Affiliations:** 1 Institute of Mathematics, University of Zurich, Zurich, Switzerland; 2 Institute of Communications and Navigation, German Aerospace Center, Oberpfaffenhofen-Wessling, Germany; 3 Vrije Universiteit Brussel, Brussels, Belgium; 4 Max-Planck-Institute for Mathematics in the Sciences, Leipzig, Germany

**Keywords:** MDPC codes, Projective bundle, Projective plane, Bit-flipping decoding algorithm, 11T71, 51E05

## Abstract

New constructions for moderate-density parity-check (MDPC) codes using finite geometry are proposed. We design a parity-check matrix for the main family of binary codes as the concatenation of two matrices: the incidence matrix between points and lines of the Desarguesian projective plane and the incidence matrix between points and ovals of a projective bundle. A projective bundle is a special collection of ovals which pairwise meet in a unique point. We determine the minimum distance and the dimension of these codes, and we show that they have a natural quasi-cyclic structure. We consider alternative constructions based on an incidence matrix of a Desarguesian projective plane and compare their error-correction performance with regards to a modification of Gallager’s bit-flipping decoding algorithm. In this setting, our codes have the best possible error-correction performance after one round of bit-flipping decoding given the parameters of the code’s parity-check matrix.

## Introduction

The close interplay between coding theory and finite geometry has emerged multiple times in the last 60 years, starting from the works of Prange [27] and Rudolph [31], where they proposed to construct linear codes starting from projective planes. Their idea was to use the incidence matrix of the plane as a generator matrix or as a parity-check matrix of a linear code, showing that the underlying geometry can be translated in metric properties of the corresponding codes. Generalizations of these constructions have been studied since the 70’s and are still the subject of active research (see [2]). The relations between these two research areas had also a strong impact in the opposite direction. The most striking example is certainly the non-existence proof of a finite projective plane of order 10 shown in [20]. This groundbreaking result came—with the help of a computer—after a series of papers analyzed the binary linear code coming from a putative projective plane of order 10.

A very important class of codes which was sensibly influenced by geometric constructions is given by low-density parity-check (LDPC) codes, which were introduced by Gallager in his seminal 1962 paper [7]. LDPC codes, as originally proposed, are binary linear codes with a very sparse parity-check matrix. This sparsity property is the bedrock of efficient decoding algorithms. Already Gallager provided two of such algorithms whose decoding complexity per iteration is linear in the block length. However, LDPC codes came to fame much later, when in 2001 Richardson, Shokrollahi and Urbanke [29] were able to show that LDPC codes are capable to approach the Shannon capacity in a practical manner. The above authors derived this result using random constructions of very large and sparse parity-check matrices. Because of these random constructions the performance of the codes was only guaranteed with high probability and there was also the practical disadvantage that the storage of a particular parity-check matrix required a lot of storage space.

There are several design parameters one wants to optimize when constructing LDPC codes. On the side of guaranteeing that the distance is reasonably large, it was realized early that it is desirable that the girth of the associated Tanner graph is large as well. This last property helps to avoid decoding failures in many decoding algorithms. Thus, in order to guarantee that an LDPC code had desirable design parameters, such as a large distance or a large girth of the associated Tanner graph, some explicit constructions were needed. Already in 1982 Margulis [23] used group theoretic methods to construct a bipartite Cayley graph whose girth was large. This line of research was extended by Rosenthal and Vontobel [30] using some explicit constructions of Ramanujan graphs, which have exceptionally large girth.

Maybe the first time objects from finite geometry were used to construct explicitly some good LDPC codes was in the work of Kou, Lin and Fossorier [19]. These authors gave four different constructions using affine and projective geometries over finite fields which did guarantee that the resulting code had a good distance and the associated Tanner graph had a girth of at least 6. Using points and lines in ${\mathbb {F}}_q^m$ Kim, Peled, Perepelitsa, Pless and Friedland [18] came up with incidence matrices representing excellent LDPC codes. In the last 15 years there has been active research to come up with further explicit constructions of LDPC codes with desirable parameters based on combinatorial structures [12, 19, 22, 35, 36].

Moderate-density parity-check (MDPC) codes were first introduced by Ouzan and Be’ery [25]. Misoczki, Tillich, Sendrier and Barreto [24] showed that MDPC codes could still be decoded with low complexity as long as the row-weight of each row vector of the parity-check matrix was not much more than the square root of the length of the code. These authors also showed that MDPC codes are highly interesting for the use in the area of code based cryptography. Similar as for LDPC codes, it is an important task to come up with explicit constructions of MDPC codes where, e.g., a good minimum distance can be guaranteed. Already Ouzan and Be’ery [25] provided a construction using cyclotomic cosets. Further constructions using quasi-cyclic codes can be found in [11, 24].

This paper adds another dowel to the theory of error-correcting codes arising from geometric objects. We propose a new construction of linear codes using projective bundles in a Desarguesian projective plane, resulting in a family of MDPC codes. Concretely, a projective bundle in a projective plane of order *q* is a collection of $q^2+q+1$ ovals which mutually intersect in a unique point. We consider the incidence structure consisting of the lines of a projective plane together with the ovals of a projective bundle. Such an incidence structure arises from studying the ${\mathbb {F}}_q$-sublines of a scattered linear set of pseudoregulus type in ${{\,\mathrm{PG}\,}}(1,q^3)$ [21]. The incidence matrix of this structure will serve as a parity-check matrix of the proposed binary codes. We completely determine their dimension and minimum distance for both *q* even and odd. In addition, we observe that we can design these codes to possess a quasi-cyclic structure of index 2. As a consequence, their encoding can be achieved in linear time and implemented with linear feedback shift registers. Moreover, also the storage space required is only half their length. We then generalize this construction and consider other variations. Their error-correcting performance with regards to Gallager’s bit-flipping algorithm is discussed.

The main motivation arises from [34], where the error-correction capability of the bit-flipping decoding algorithm on the parity-check matrix of an MDPC code was analyzed. There, it was derived that its performance is inversely proportional to the maximum column intersection of the parity-check matrix, which is the maximum number of positions of ones that two distinct columns share. We show indeed that the maximum column intersection of the derived parity-check matrices is the smallest possible for the chosen parameters, implying in turn the best possible performance for one round of the bit-flipping algorithm.

The paper is organized as follows: Sect. 2 consists of the coding theory background needed in the paper. In particular, we introduce the family of MDPC codes and we recall the result on the performance of the bit-flipping algorithm presented in [34], which was decisive for the idea of this construction. In Sect. 3 we give a brief overview on projective planes, studying the basic properties of codes arising from them. Section 4 is dedicated to the new proposed MDPC code design using projective bundles. Here, we study some of the code properties and we determine its dimension, minimum distance and minimum weight codewords. The paper is based on the master’s thesis of the first author [5] and in this section we extend the results which were originally stated there. The goal of Sect. 5 is to generalize the results stated in Sect. 4 in order to have more flexibility in the choice of the parameters. This is done by using several disjoint projective bundles instead of only one. We then propose another construction of binary codes in Sect. 6, which only uses the incidence matrix of a projective plane and its transpose, and study minimum distance and minimum weight codewords. We then compare the error-correction performances of the new codes by running several experiments. Finally, we recap our findings and draw some conclusive remarks in Sect. 7.

## Coding theory and moderate-density parity-check codes

Let us start by briefly recalling some basics of coding theory. Throughout the paper *q* will always be a prime power, and we will denote the finite field with *q* elements by ${\mathbb {F}}_q$. The set of vectors of length *n* over ${\mathbb {F}}_q$ will be denoted by ${\mathbb {F}}_q^n$.

We consider the *Hamming weight* on ${\mathbb {F}}_q^n$ defined as

$$\begin{aligned} {{\,\mathrm{wt}\,}}(v):=\left| {\lbrace i \in \{1,\ldots ,n\} \mid v_i\ne 0\rbrace }\right| . \end{aligned}$$

It is well-known that it induces a metric, namely the *Hamming distance* which is given by

$$\begin{aligned} \begin{array}{rccl} {{\,\mathrm{d}\,}}_{{{\,\mathrm{H}\,}}}: &{} {\mathbb {F}}_q^n \times {\mathbb {F}}_q^n &{} \longrightarrow &{} {\mathbb {N}}\\ &{} (u,v) &{} \longmapsto &{} {{\,\mathrm{wt}\,}}(u-v). \end{array} \end{aligned}$$

### Definition 2.1

A *q*-*ary linear code* *C* of length *n* and dimension $\dim (C) = k$ is a *k*-dimensional linear subspace of ${\mathbb {F}}_q^n$ endowed with the Hamming metric. The *minimum distance* of *C* is the minimum among all the possible weights of the nonzero codewords and it is denoted by ${{\,\mathrm{d}\,}}(C)$, i.e.

$$\begin{aligned} {{\,\mathrm{d}\,}}(C) := \min \lbrace {{\,\mathrm{wt}\,}}(c) \, | \, c \in C, \, c \not = 0 \rbrace . \end{aligned}$$

In general, finding the minimum distance of a linear code and classifying its nonzero codewords of minimum weight is not an easy task. Even for linear codes from geometric constructions, it is often highly non-trivial to find sharp bounds or a classification of the smallest weight words, see for example [1, 3, 17, 26, 35].

A *q*-ary linear code of length *n* and dimension *k* will be denoted for brevity by $[n,k]_q$ code, or by $[n,k,d]_q$ code if the minimum distance *d* is known.

Any $[n,k]_q$ code *C* has a dual code which is defined as

$$\begin{aligned} C^\perp = \lbrace x \in {\mathbb {F}}_q^n \, | \, x\cdot c^\top = 0, \, \forall c \in C \rbrace . \end{aligned}$$

A *generator matrix* of an $[n,k]_q$ code *C* is a matrix $G\in {\mathbb {F}}_q^{k\times n}$ whose rows form a basis of *C*. A generator matrix $H \in {\mathbb {F}}_q^{(n-k)\times n}$ for the dual code $C^\perp$ is called a *parity-check matrix* of *C*. Note that *C* can also be represented by a parity-check matrix *H*, since it corresponds to its right kernel, i.e.

$$\begin{aligned} C = \ker (H) = \lbrace c \in {\mathbb {F}}_q^n \, | \, c\cdot H^\top = 0 \rbrace . \end{aligned}$$

A matrix $A\in {\mathbb {F}}_q^{r\times s}$ is said to have *row-weight* *w*, for some nonnegative integer *w*, if every row of *A* has Hamming weight equal to *w*. Similarly, we say that *A* has *column-weight* *v*, if each of its columns has Hamming weight *v*.

In the following we will focus on the family of moderate-density parity-check (MDPC) codes. They are an extension of the well-known low density parity-check (LDPC) codes, and they are defined by the row-weight of a parity-check matrix. The terminology was first introduced in [25], and then these codes were reintroduced and further generalized in [24] for cryptographic purposes.

### Definition 2.2

Let $\{C_i\}$ be a family of binary linear codes of length $n_i$ with parity-check matrix $H_i$. If $H_i$ has row weight $\mathcal {O}(\sqrt{n_i})$, $\{C_i\}$ is called a (family of) *moderate-density parity-check (MDPC)* code. If, in addition, the weight of every column of $H_i$ is a constant $v_i$ and the weight of every row of the $H_i$ is a constant $w_i$ we say the MDPC code is of type $(v_i, w_i)$.

MDPC codes have been constructed in various ways. In their seminal paper [25], Ouzan and Be’ery designed cyclic MDPC codes carefully choosing the idempotent generator of the dual code. This structure has been generalized in order to design quasi-cyclic MDPC codes (see [11, 24]). A different approach has been proposed in [34], where a random model is considered.

In the definition of an MDPC code the chosen parity-check matrix is very important. Indeed, as for LDPC codes, an MDPC code automatically comes together with a decoding algorithm—for instance the bit-flipping algorithm—whose performance depends on the chosen parity-check matrix. Thus, in order to study the error-correction performance, we introduce the following quantity.

### Definition 2.3

Let *H* be a binary matrix. The *maximum column intersection* is the maximal cardinality of the intersection of the supports of any pair of distinct columns of *H*.

The following result was found by Tillich in 2018 (for more details and the proof see [34]). It states the amount of errors that can be corrected within one round of the bit-flipping decoding algorithm.

### Theorem 2.4

Let *C* be an MDPC code of type (*v*, *w*) with parity-check matrix *H*. Let $s_H$ denote the maximum column intersection of *H*. Performing one round of the bit-flipping decoding algorithm with respect to *H*, we can correct all errors of weight at most $\lfloor \frac{v}{2 \cdot s_H}\rfloor$.

It hence follows that, the smaller $s_H$, the more errors can be corrected after one round of the bit-flipping decoding algorithm. Using a random construction as the one proposed by Tillich, the expected value for the maximum column intersection $s_H$ is ${\mathcal {O}}(\frac{\log n}{\log \log n})$, as shown in [34, Proposition 2]. We would like to design MDPC codes in such a way that $s_H$ is as small as possible and, more importantly, that $s_H$ is deterministic. With this we can ensure that the bit-flipping decoder is able to correct a given amount of errors, which we will discuss in Sects. 4.3 and 6.2.

## MDPC codes from projective planes

The projective plane ${{\,\mathrm{PG}\,}}(2,q)$ is a point-line geometry constructed from a three-dimensional vector space *V* over ${\mathbb {F}}_q$. Its points and lines are the one- and two-dimensional subspaces of *V*, respectively and the containment relation in *V* defines the incidence relation of the plane. It has $q^2+q+1$ points and equally many lines. The geometry satisfies the following properties:

1. Any two distinct points are incident with exactly one common line;
2. Any two distinct lines are incident with exactly one common point;
3. There are four points such that no three of them are collinear.

This means that ${{\,\mathrm{PG}\,}}(2,q)$ can also be regarded as a symmetric 2-$(q^2+q+1,q+1,1)$-design, where the lines correspond to the blocks. Moreover, every line in ${{\,\mathrm{PG}\,}}(2,q)$ is incident with $q+1$ points and dually, every point is incident with $q+1$ lines. One way to represent ${{\,\mathrm{PG}\,}}(2,q)$ is by an incidence matrix. This is a matrix *A* whose rows and columns are indexed by points and lines, respectively such that

$$\begin{aligned} (A)_{p\ell } = {\left\{ \begin{array}{ll} 1 \text { if } p \text { is incident with } \ell \\ 0 \text { otherwise.} \end{array}\right. } \end{aligned}$$

Here we describe an alternative way to represent the projective plane ${{\,\mathrm{PG}\,}}(2,q)$. We can identify the set of points with the integers modulo $q^2+q+1$. For the description of the lines, we will follow the instruction presented by Hirschfeld in [10, p77—p79]. Let us therefore introduce the following set.

### Definition 3.1

A set $D=\{d_0, \dots , d_r\}\subseteq {\mathbb {Z}}/(r^2+r+1){\mathbb {Z}}$ is called a *perfect difference set*, if all differences $(d_i - d_j)$ with $i \not = j$, are distinct modulo $r^2+r+1$, for $i, j \in \{0, \dots , r\}$.

### Example 3.2

For instance, consider $r=2$. One can show that the set $D = \{0, 1, 3\}$ of $r+1 = 3$ integers is indeed a perfect difference set, since any two differences between two distinct elements are pairwise disjoint modulo $r^2+r+1 = 7$.

Hirschfeld showed in [10, Theorem 4.2.2 and its Corollary] that the set of lines of ${{\,\mathrm{PG}\,}}(2, q)$ is fully described by the circulant shifts modulo $q^2+q+1$ of a perfect difference set of $q+1$ elements. In this way we obtain a circulant incidence matrix in which the support of the first column is *D*.

In order to illustrate this, consider the Fano plane ${{\,\mathrm{PG}\,}}(2, 2)$ consisting of seven points and lines. We have seen, that the points will be identified with the integers modulo $q^2+q+1 = 7$. For the set of lines we will use the cyclic shifts (modulo 7) of the set $D = \{ 0, 1, 3 \}$, which we have seen is in fact a perfect difference set. Explicitly, we obtain the following set of points $\mathcal {P}$ and set of lines $\mathcal {L}$

$$\begin{aligned} \mathcal {P}&= \{ 0, 1, 2, 3, 4, 5, 6 \}, \\ \mathcal {L}&= \lbrace \{ 0+i, 1+i, 3+i \} \, | \, i \in \{0, \ldots , 6 \} \rbrace . \end{aligned}$$

The defining properties of projective planes have made them a good source of error-correcting codes by taking their incidence matrices as the parity-check matrix, as was done already in the late 1950s, cf. [27] or [31].

### Definition 3.3

Let *H* be an incidence matrix of $\Uppi = {{\,\mathrm{PG}\,}}(2,q)$ over the binary finite field ${\mathbb {F}}_2$. We define the code $C_2(\Uppi )^\perp \subseteq {\mathbb {F}}_2^{q^2+q+1}$ via

$$\begin{aligned} C_2(\Uppi )^{\perp } =\ker (H). \end{aligned}$$

Codes from planes have been intensively studied and many properties have been derived thanks to the underlying geometric structure. Among the most relevant properties, Graham and MacWilliams [9] completely determined the dimension of the codes $C_p(\Uppi )^\perp$ over ${\mathbb {F}}_p$ and their minimum distance when $p=2$ was determined by Assmus and Key [2]. Here we state the two results, restricting ourselves only to the case $p=2$.

### Theorem 3.4

The code $C_2(\Uppi )^\perp$ is a $[q^2+q+1,k,d]_2$ code, where

$$\begin{aligned} (k,d)={\left\{ \begin{array}{ll} (1,q^2+q+1) &{} \text{ if } q \text{ is } \text{ odd } , \\ (2^{2h}-3^h+2^h, 2^h+2) &{} \text{ if } q=2^h. \end{array}\right. } \end{aligned}$$

The first part just follows from the observation that if *A* is the incidence matrix of a projective plane of order *q*, then by definition

$$\begin{aligned}A^\top \!A = AA^\top = qI + J,\end{aligned}$$

where *I* is the identity matrix and *J* the all-one matrix of size $q^2+q+1$.

From Theorem 3.4 we can see that binary codes from ${{\,\mathrm{PG}\,}}(2,q)$ are only interesting whenever *q* is even. Moreover, one can see that the incidence matrix of $\Uppi$ has constant row and column weight equal to $q+1$ which is $\mathcal O(\sqrt{q^2+q+1})$. Hence, codes from projective planes are very special examples of MDPC codes. With the aid of Theorem 2.4, we can show that one round of the bit-flipping algorithm on these codes permits to decode up to half the minimum distance with no failure probability, for any projective plane.

### Theorem 3.5

Let $\Uppi$ be a projective plane of even order and *H* its incidence matrix, which is the parity-check matrix of the code $C_2(\Uppi )^\perp$. After performing one round of bit-flipping on *H* we can correct any error of weight up to $\lfloor \frac{d-1}{2}\rfloor$, where *d* is the minimum distance of $C_2(\Uppi )^\perp$.

### Proof

Since a projective plane is in particular a symmetric 2-$(q^2+q+1,q+1,1)$-design, the maximum column intersection of *H* is 1. Moreover, the matrix *H* is of type $(q+1,q+1)$. Hence, applying Theorem 2.4, we obtain that one round of the bit-flipping algorithm corrects every error of weight at most $\lfloor \frac{d-1}{2}\rfloor$.$\square$

Theorem 3.5 shows that codes from planes are really powerful, and have the best performance according to Theorem 2.4, for a given matrix of type $(q+1,q+1)$ and size $(q^2+q+1)\times (q^2+q+1)$. However, we can only construct codes from projective planes of even order, resulting in $[2^{2h}+2^h+1,2^{2h}-3^h+2^h, 2^h+2]_2$ codes. This lack of choice of the parameters motivated many variations on this construction. In the last 50 years, many codes have been constructed based on underlying geometric objects: Euclidean and projective geometries over finite fields [6, 19, 33], linear representation of Desarguesian projective planes [26], (semi-)partial geometries [16, 35], generalized quadrangles [17, 36], generalized polygons [22], Ramanujan graphs [23, 30], *q*-regular bipartite graphs from point line geometries [18] and other incidence structures coming from combinatorial designs [12–15].

For the same reason, we propose a new construction of (families of) MDPC codes based on a suitable system of conics in a Desarguesian projective plane that behaves itself like a projective plane. This is encapsulated in the concept of *projective bundles*, which we define, along with other notions from finite geometry, in the following section.

## MDPC codes from projective bundles

In this section we present the new MDPC codes using projective bundles by constructing its parity-check matrix. We start off by introducing the relevant geometrical objects, which are ovals and projective bundles in ${{\,\mathrm{PG}\,}}(2,q)$.

### Definition 4.1

An *oval* in ${{\,\mathrm{PG}\,}}(2,q)$ is a set of $q+1$ points, such that every line intersects it in at most two points.

The classical example of an oval is a non-degenerate conic, i.e. the locus of an irreducible homogeneous quadratic equation. When *q* is odd, Segre’s seminal result [32] shows that the converse is also true: every oval is a conic.

### Definition 4.2

A line in ${{\,\mathrm{PG}\,}}(2,q)$ is *skew*, *tangent* or *secant* to a given oval if it intersects it in zero, one or two points, respectively.

We recall some properties of ovals which were first recorded by Qvist [28]. We include the proof as it will be relevant later.

### Lemma 4.3

An oval in ${{\,\mathrm{PG}\,}}(2,q)$ has $q+1$ tangent lines, one in each point.

- If *q* is odd, every point not on the oval is incident with zero or two tangent lines.
- If *q* is even, then all tangent lines are concurrent.

### Proof

Consider a point on the oval. Then there are *q* lines through this point intersecting the oval in one more point. This means that one line remains, which is necessarily a tangent line, hence proving the first part of the lemma.

Now suppose that *q* is odd and consider a point on a tangent line, not on the oval. As the number of points of the oval, i.e. $q+1$, is even, this point is incident with an odd number of tangent lines more. Since the point is arbitrary, and there are $q+1$ tangent lines in total, this implies that every point on the tangent line (but not on the oval) is incident with exactly two tangent lines.

When *q* is even, we consider a point on a secant line, but not on the oval and proceed in a similar fashion as before: the number of points on the oval but not on the secant is $q-1$ and hence odd, so this point is incident with an odd number of tangents. Since this point is arbitrary, and there are $q+1$ tangent lines, this implies that every point on the secant line is incident with exactly one tangent line. This also means that the intersection point of two tangent lines is necessarily the intersection of all tangent lines: this intersection point cannot be on a secant as we just saw, so the $q+1$ lines through the point are either tangent or skew. Since the oval has $q+1$ points, which are all contained in one of these lines, we deduce that they must be all tangent.$\square$

When *q* is even, one can add the point of concurrency of the tangent lines, which is called the *nucleus*, to the oval to obtain a set of $q+2$ points that has zero or two points in common with every line. This leads us to the following definition.

### Definition 4.4

A *hyperoval* is a set of $q+2$ points in ${{\,\mathrm{PG}\,}}(2,q)$ such that every line has zero or two points in common. A *dual hyperoval* is a set of $q+2$ lines such that every point is incident with zero or two lines.

We will encounter these objects again later on. We are now in the position to define projective bundles.

### Definition 4.5

A *projective bundle* is a collection of $q^2+q+1$ ovals of ${{\,\mathrm{PG}\,}}(2,q)$ mutually intersecting in a unique point.

Projective bundles were introduced by Glynn in his Ph.D. thesis [8] under the name ‘packings of $(q+1)$-arcs’. The original definition is a bit more general and applies to any projective plane instead of just ${{\,\mathrm{PG}\,}}(2,q)$. Since the only known projective bundles exist in ${{\,\mathrm{PG}\,}}(2,q)$, it suffices for our purposes to restrict ourselves to this case.

It follows from the definition that one can consider the points of ${{\,\mathrm{PG}\,}}(2,q)$ and the ovals of a projective bundle as the points and lines of a projective plane of order *q*. We can then define the notion of secant, tangent and skew ovals (which belong to the projective bundle) with respect to a line. Moreover, one can interchange the role of lines and ovals in the proof of Lemma 4.3 and find the following statement, which we record for convenience.

### Lemma 4.6

Given a projective bundle, a line in ${{\,\mathrm{PG}\,}}(2,q)$ has $q+1$ tangent ovals, one in each point.

- If *q* is even, then all tangent ovals are concurrent.
- If *q* is odd, every point not on the line is incident with zero or two tangent ovals to this line.

When *q* is even, we can similarly as before define a *hyperoval of ovals* as a set of $q+2$ ovals such that every point is contained in zero or two of them.

An interesting property of projective bundles is that a third projective plane can be found. This result is due to Glynn [8, Theorem 1.1.1] and served as the motivation for projective bundles: to possibly find new projective planes from known ones.

### Theorem 4.7

Consider the ovals of a projective bundle and the lines of ${{\,\mathrm{PG}\,}}(2,q)$ as points and lines, respectively, with incidence defined by tangency. Then this point-line geometry is a projective plane of order *q*.

We can rephrase this in terms of incidence matrices as follows: if *A* and *B* are the point-line incidence matrices of ${{\,\mathrm{PG}\,}}(2,q)$ and the projective plane whose lines are the ovals of a projective bundle, then $A^\top B \!\pmod {2}$ is again the incidence matrix of a projective plane. However, for *q* even this idea to construct new projective planes does not work, since then all three projective planes are isomorphic [8, Corollary 1.1.1].

Glynn showed that projective bundles indeed exist for any *q*, and his examples are all bundles of conics. When *q* is odd, he showed the existence of three distinct types of projective bundles in ${{\,\mathrm{PG}\,}}(2,q)$, by identifying them with planes in ${{\,\mathrm{PG}\,}}(5,q)$. It was shown in [4] that perfect difference sets can also be used to describe these projective bundles. In fact, given a perfect difference set $D\subseteq {\mathbb {Z}}/(q^2+q+1){\mathbb {Z}}$ and its circular shifts corresponding to the set of lines of ${{\,\mathrm{PG}\,}}(2, q)$, the three bundles are represented in the following way.

1. *Cirumscribed bundle:* set of all circular shifts of $-D$.
2. *Inscribed bundle:* set of all circular shifts of 2*D*.
3. *Self-polar bundle:* set of all circular shifts of *D*/2.

We are now going to construct the parity-check matrix as mentioned at the beginning of this section. Let us denote the projective plane formed by the points and lines of ${{\,\mathrm{PG}\,}}(2,q)$ by $\Uppi$ and the one formed by the points and the ovals of a projective bundle of ${{\,\mathrm{PG}\,}}(2,q)$ by $\Upgamma$. Then define

1 $$\begin{aligned} H = ( \,A \, \mid \, B\,), \end{aligned}$$

where *A* and *B* are the incidence matrices of $\Uppi$ and $\Upgamma$, respectively. Hence, we obtain a $(q^2+q+1)\times 2(q^2+q+1)$ binary matrix defined by the points, lines and ovals of a projective bundle of ${{\,\mathrm{PG}\,}}(2,q)$.

### Definition 4.8

A binary linear code with parity-check matrix *H* given in (1) is called a *projective bundle code* and we will denote it by

$$\begin{aligned} C_2(\Uppi \sqcup \Upgamma )^\perp = \ker (H). \end{aligned}$$

Clearly, the matrix *H* given in (1) has constant row-weight $w = 2(q+1)$ and constant column-weight $v = q+1$. Hence, $C_2(\Uppi \sqcup \Upgamma )^\perp$ is an MDPC code of length $n=2(q^2+q+1)$ and type $(q+1, 2(q+1))$.

### Remark 4.9

The family of MDPC codes that we are considering is built upon a parity-check matrix as in (1). In such a matrix the number of columns is twice the number of rows and this coincides with the setting originally studied in [24].

### Example 4.10

Let us give a short example of a projective bundle code for a relatively small parameter $q = 3$. Hence, we consider the projective plane ${{\,\mathrm{PG}\,}}(2, 3)$. Recall, that the set of points $\mathcal {P}$ is given by the set of integers modulo $q^2+q+1 = 13$. The set of lines $\mathcal {L}$ is defined by the image of a perfect difference set *D* of four integers under repeated application of the Singer cycle $S(i) = i+1$. It is easy to verify that $D = \{ 0, 1, 3, 9 \}$ is a perfect difference set, i.e.

$$\begin{aligned} \mathcal {L} = \lbrace \{ 0+i, 1+i, 3+i, 9 + i\} \, | \, i \in {\mathbb {Z}}/13{\mathbb {Z}}\rbrace . \end{aligned}$$

At this point, let us choose an inscribed bundle $\mathcal {B}_I$ in ${{\,\mathrm{PG}\,}}(2, 3)$. As shown above, this bundle is represented by the cyclic shifts of $2D = \lbrace 0, 2, 5, 6 \rbrace$. Hence, we obtain

$$\begin{aligned} \mathcal {B}_I = \lbrace \{ 0+i, 2+i, 5+i, 6 + i\} \, | \, i \in {\mathbb {Z}}/13{\mathbb {Z}}\rbrace . \end{aligned}$$

Concatenating the two corresponding incidence matrices *A* and *B* yields the desired parity-check matrix

$$\begin{aligned} H = \left( \begin{array}{ccccccccccccc|ccccccccccccc} 1 &{} \cdot &{} \cdot &{} \cdot &{} 1 &{} \cdot &{} \cdot &{} \cdot &{} \cdot &{} \cdot &{} 1 &{} \cdot &{} 1 &{} 1 &{} \cdot &{} \cdot &{} \cdot &{} \cdot &{} \cdot &{} \cdot &{} 1 &{} 1 &{} \cdot &{} \cdot &{} 1 &{} \cdot \\ 1 &{} 1 &{} \cdot &{} \cdot &{} \cdot &{} 1 &{} \cdot &{} \cdot &{} \cdot &{} \cdot &{} \cdot &{} 1 &{} \cdot &{} \cdot &{} 1 &{} \cdot &{} \cdot &{} \cdot &{} \cdot &{} \cdot &{} \cdot &{} 1 &{} 1 &{} \cdot &{} \cdot &{} 1 \\ \cdot &{} 1 &{} 1 &{} \cdot &{} \cdot &{} \cdot &{} 1 &{} \cdot &{} \cdot &{} \cdot &{} \cdot &{} \cdot &{} 1 &{} 1 &{} \cdot &{} 1 &{} \cdot &{} \cdot &{} \cdot &{} \cdot &{} \cdot &{} \cdot &{} 1 &{} 1 &{} \cdot &{} \cdot \\ 1 &{} \cdot &{} 1 &{} 1 &{} \cdot &{} \cdot &{} \cdot &{} 1 &{} \cdot &{} \cdot &{} \cdot &{} \cdot &{} \cdot &{} \cdot &{} 1 &{} \cdot &{} 1 &{} \cdot &{} \cdot &{} \cdot &{} \cdot &{} \cdot &{} \cdot &{} 1 &{} 1 &{} \cdot \\ \cdot &{} 1 &{} \cdot &{} 1 &{} 1 &{} \cdot &{} \cdot &{} \cdot &{} 1 &{} \cdot &{} \cdot &{} \cdot &{} \cdot &{} \cdot &{} \cdot &{} 1 &{} \cdot &{} 1 &{} \cdot &{} \cdot &{} \cdot &{} \cdot &{} \cdot &{} \cdot &{} 1 &{} 1 \\ \cdot &{} \cdot &{} 1 &{} \cdot &{} 1 &{} 1 &{} \cdot &{} \cdot &{} \cdot &{} 1 &{} \cdot &{} \cdot &{} \cdot &{} 1 &{} \cdot &{} \cdot &{} 1 &{} \cdot &{} 1 &{} \cdot &{} \cdot &{} \cdot &{} \cdot &{} \cdot &{} \cdot &{} 1 \\ \cdot &{} \cdot &{} \cdot &{} 1 &{} \cdot &{} 1 &{} 1 &{} \cdot &{} \cdot &{} \cdot &{} 1 &{} \cdot &{} \cdot &{} 1 &{} 1 &{} \cdot &{} \cdot &{} 1 &{} \cdot &{} 1 &{} \cdot &{} \cdot &{} \cdot &{} \cdot &{} \cdot &{} \cdot \\ \cdot &{} \cdot &{} \cdot &{} \cdot &{} 1 &{} \cdot &{} 1 &{} 1 &{} \cdot &{} \cdot &{} \cdot &{} 1 &{} \cdot &{} \cdot &{} 1 &{} 1 &{} \cdot &{} \cdot &{} 1 &{} \cdot &{} 1 &{} \cdot &{} \cdot &{} \cdot &{} \cdot &{} \cdot \\ \cdot &{} \cdot &{} \cdot &{} \cdot &{} \cdot &{} 1 &{} \cdot &{} 1 &{} 1 &{} \cdot &{} \cdot &{} \cdot &{} 1 &{} \cdot &{} \cdot &{} 1 &{} 1 &{} \cdot &{} \cdot &{} 1 &{} \cdot &{} 1 &{} \cdot &{} \cdot &{} \cdot &{} \cdot \\ 1 &{} \cdot &{} \cdot &{} \cdot &{} \cdot &{} \cdot &{} 1 &{} \cdot &{} 1 &{} 1 &{} \cdot &{} \cdot &{} \cdot &{} \cdot &{} \cdot &{} \cdot &{} 1 &{} 1 &{} \cdot &{} \cdot &{} 1 &{} \cdot &{} 1 &{} \cdot &{} \cdot &{} \cdot \\ \cdot &{} 1 &{} \cdot &{} \cdot &{} \cdot &{} \cdot &{} \cdot &{} 1 &{} \cdot &{} 1 &{} 1 &{} \cdot &{} \cdot &{} \cdot &{} \cdot &{} \cdot &{} \cdot &{} 1 &{} 1 &{} \cdot &{} \cdot &{} 1 &{} \cdot &{} 1 &{} \cdot &{} \cdot \\ \cdot &{} \cdot &{} 1 &{} \cdot &{} \cdot &{} \cdot &{} \cdot &{} \cdot &{} 1 &{} \cdot &{} 1 &{} 1 &{} \cdot &{} \cdot &{} \cdot &{} \cdot &{} \cdot &{} \cdot &{} 1 &{} 1 &{} \cdot &{} \cdot &{} 1 &{} \cdot &{} 1 &{} \cdot \\ \cdot &{} \cdot &{} \cdot &{} 1 &{} \cdot &{} \cdot &{} \cdot &{} \cdot &{} \cdot &{} 1 &{} \cdot &{} 1 &{} 1 &{} \cdot &{} \cdot &{} \cdot &{} \cdot &{} \cdot &{} \cdot &{} 1 &{} 1 &{} \cdot &{} \cdot &{} 1 &{} \cdot &{} 1 \\ \end{array}\right) , \end{aligned}$$

where the zero entries in the parity-check matrix are represented by dots.

### Remark 4.11

Observe that the matrix *H* defined in (1) can be constructed from a perfect difference set *D*, by taking the circular shifts of *D* and *sD*, with $s \in \{-1,2,2^{-1}\}$. Such a matrix has a double circulant structure. Thus, the resulting code $C_2(\Uppi \sqcup \Upgamma )^\perp$ is quasi-cyclic of index 2, and encoding can be achieved in linear time and implemented with linear feedback shift registers. Furthermore, we can also deduce—because of the circular structure—that the number of bits required to describe the parity-check matrix is about $(q+1) \log _2(q^2+q+1)$, which is approximately $2 (q+1) \log _2(q)$.

### Remark 4.12

For *q* odd, when $\Upgamma$ is a circumscribed bundle, the incidence structure $\Uppi \sqcup \Upgamma$ given by the points of ${{\,\mathrm{PG}\,}}(2,q)$ and the set of lines together with the ovals in $\Upgamma$ has already appeared in the literature. Indeed, it coincides with the incidence structure given by a scattered linear set of pseudoregulus type in ${{\,\mathrm{PG}\,}}(1,q^3)$ and the set of its ${\mathbb {F}}_q$-sublines; see [21, Remark 20].

In the following subsections we will analyse the dimension, minimum distance and error-correction performance with respect to the bit-flipping decoding algorithm of $C_2(\Uppi \sqcup \Upgamma )^\perp$.

### Dimension

Recall from Theorem 3.4 that a binary code $C_2(\Uppi )^\perp$ from a projective plane $\Uppi \cong {{\,\mathrm{PG}\,}}(2,q)$, is either trivial of codimension 1—when *q* is odd—or it is non-trivial to determine its dimension—when *q* is even. In our case, the structure of our code allows to both have a non-trivial code and to determine the exact dimension for all *q*. To do so, recall that if *A* is the incidence matrix of a projective plane of order *q*, then

$$\begin{aligned}AA^\top = A^\top \!A = qI + J,\end{aligned}$$

where *J* is the all-one matrix of appropriate size.

Using this result we are able to state the dimension of $C_2(\Uppi \sqcup \Upgamma )^\perp$.

#### Proposition 4.13

Let $\Uppi$ be a projective plane of order *q* and let $\Upgamma$ be a projective bundle in $\Uppi$. Then,

$$\begin{aligned} \dim \left( C_2(\Uppi \sqcup \Upgamma )^\perp \right) = {\left\{ \begin{array}{ll} q^2+q+2 &{} \text{ if } q \text{ is } \text{ odd, } \\ 2^{2h+1}+2^{h+1}-2(3^h)+1 &{} \text{ if } q=2^h. \end{array}\right. } \end{aligned}$$

#### Proof

In order to determine the dimension of the code, we need to compute the rank of a parity-check matrix $H = ( \,A \, \mid \, B\,)$. Since *H* is of size $(q^2+q+1)\times 2(q^2+q+1)$, we can already say that the rank of *H* is at most $q^2+q+1$. Now we consider the two cases.

**Case I** ${\underline{q}}$ **odd** We know from Theorem 3.4 that ${{\,\mathrm{rk}\,}}(A) = q^2+q$, which gives us the lower bound ${{\,\mathrm{rk}\,}}(H) \ge {{\,\mathrm{rk}\,}}(A) = q^2+q$.

The matrix *H* has full rank $q^2+q+1$ if and only if there exists no element in the left-kernel, i.e. if there is no nonzero vector $x\in {\mathbb {F}}_2^{q^2+q+1}$ such that

2 $$\begin{aligned} x H = 0. \end{aligned}$$

However, if *x* is the all-one vector then Eq. (2) is satisfied. Hence, there is an element in the cokernel which implies that *H* cannot have full rank and we conclude that $\dim C_2(\Uppi \sqcup \Upgamma )^\perp = q^2+q+2$.

**Case II** ${\underline{q}}$ **even** In this case, we consider the matrix

$$\begin{aligned} H^\top \!H=\begin{pmatrix} A^\top \! A \;&{} A^\top \!B\\ B^\top \!A \;&{} B^\top \!B \end{pmatrix}=\begin{pmatrix} J &{} A^\top \!B\\ (A^\top \!B)^\top &{} J \end{pmatrix}. \end{aligned}$$

By Theorem 4.7 and the discussion below, $A^\top \!B = C$ is again the incidence matrix of ${{\,\mathrm{PG}\,}}(2,q)$, and hence the sum of all its rows/columns is equal to the all-one vector. Therefore, by doing row operations on $H^\top \!H$, we obtain the matrix

$$\begin{aligned} \begin{pmatrix} 0 &{} A^\top \!B+J\\ (A^\top \!B)^\top &{} J \end{pmatrix}, \end{aligned}$$

which has the same rank as $H^\top \!H$. Hence,

$$\begin{aligned} {{\,\mathrm{rk}\,}}(H)\ge {{\,\mathrm{rk}\,}}(H^\top \!H)={{\,\mathrm{rk}\,}}(A^\top \!B)+{{\,\mathrm{rk}\,}}(A^\top \!B+J)\ge 2{{\,\mathrm{rk}\,}}(A^\top \!B)-1, \end{aligned}$$

where the last inequality comes from the fact that *J* has rank 1, and the rank satisfies the triangle inequality. On the other hand, we have that the all-one vector is in the column spaces of both *A* and *B*, showing that ${{\,\mathrm{rk}\,}}(H)\le {{\,\mathrm{rk}\,}}(A)+{{\,\mathrm{rk}\,}}(B)-1$. Since *A*, *B* and $A^\top \!B$ are all incidence matrices of a Desarguesian plane, they all have the same rank. Therefore, combining the two inequalities, we obtain

$$\begin{aligned} {{\,\mathrm{rk}\,}}(H)=2{{\,\mathrm{rk}\,}}(A)-1, \end{aligned}$$

and using Theorem 3.4, we can conclude that

$$\begin{aligned} \dim \left( C_2(\Uppi \sqcup \Upgamma )^\perp \right)&=2(q^2+q+1)-{{\,\mathrm{rk}\,}}(H)=2(q^2+q+1)-2{{\,\mathrm{rk}\,}}(A)+1\\&=2\dim (C_2(\Uppi )^\perp ))+1=2^{2h+1}+2^{h+1}-2(3^h)+1. \end{aligned}$$

$$\square $$

We can thus already say that $C_2(\Uppi \sqcup \Upgamma )^\perp$ is a $[2(q^2+q+1), q^2+q+2]_2$ MDPC code of type $(q+1,2q+1)$.

### Minimum distance

As mentioned earlier, we are interested in the error-correction capability. A relevant quantity to give information about error-correction and also error-detection is the minimum distance of a linear code.

In the following we will determine the exact value of the minimum distance of $C_2(\Uppi \sqcup \Upgamma )^\perp$. An important observation for the proof is that geometrically, the support of a codeword of $C_2(\Uppi \sqcup \Upgamma )^\perp$ corresponds to a set of lines and ovals such that every point of ${{\,\mathrm{PG}\,}}(2,q)$ is covered an even number of times.

#### Theorem 4.14

The minimum distance of $C_2(\Uppi \sqcup \Upgamma )^\perp$ is $q+2$ and the supports of the minimum weight codewords can be characterized, depending on the parity of *q*. For *q* odd, the support of a minimum weight codeword is

- an oval and its $q+1$ tangent lines, or
- a line and its $q+1$ tangent ovals.

On the other hand for *q* even, we find that the support of a minimum weight codeword is

- a dual hyperoval, or
- a hyperoval of ovals.

#### Proof

Take a codeword of minimum weight in $C_2(\Uppi \sqcup \Upgamma )^\perp$ and consider its support. This is a set of *r* lines *L* and *s* ovals *O* such that every point in ${{\,\mathrm{PG}\,}}(2,q)$ is incident with an even number of these elements. We will show that $r+s \ge q+2$ and equality only holds for the two examples stated.

Every point is incident with $q+1$ lines and $q+1$ conics, so let $a_i$, $0 \le i \le 2q+2$, be the number of points that are covered *i* times by the *r* lines and *s* conics in the support of the minimum weight codeword. Then we can double count the tuples $(P), (P,E_1), (P,E_1,E_2)$, where *P* is a point and $E_1,E_2 \in L \cup O$ are lines or ovals incident with this point. Remark that by assumption $a_i = 0$ whenever *i* is odd. We find the following three expressions:

3 $$\begin{aligned} \sum _{i=0}^{2q+2} a_i&= q^2+q+1 \end{aligned}$$

4 $$\begin{aligned} \sum _{i=0}^{2q+2} ia_i&= (r+s)(q+1) \end{aligned}$$

5 $$\begin{aligned} \sum _{i=0}^{2q+2} i(i-1)a_i&\le r(r-1)+s(s-1)+2rs, \end{aligned}$$

where the last inequality follows as a line and oval intersect in at most two points. From these equations, we can find $\sum _{i=0}^{2q+2}i(i-2) a_i \le (r+s)(r+s-q-2)$ and hence $r+s \ge q+2$, as the sum on the left-hand side has only non-negative terms. Moreover, in the case of equality, $a_i = 0$ whenever $i \notin \{0,2\}$.

Now consider a codeword of weight $r+s = q+2$, consisting of *r* lines *L* and *s* ovals *O*. We will investigate the cases *q* odd and even separately and show the characterisation.

**Case I** ${\underline{q}}$ **odd** Since $q+2$ is odd and hence one of *r* or *s* is, we can suppose without loss of generality that *r* is odd. The argument works the same when *s* is odd, by interchanging the roles of lines and ovals.

Consider a line not in *L*. Then this line is intersected an odd number of times by the *r* lines in *L*. Therefore, it should be tangent to an odd number of ovals in *O*, recalling that every point is incident with zero or two elements from $L \cup O$. In particular, any line not in *L* is tangent to at least one oval in *O*. So count the *N* pairs $(\ell ,c)$, where $\ell$ is a line not in *L*, $c \in O$ and $|\ell \cap O| = 1$. By the previous observation, it follows that $q^2+q+1-r = q^2-1+s \le N$. On the other hand, a oval has $q+1$ tangent lines so that $N \le s(q+1)$. Combining these two leads to $s \ge q$, which implies that $r = 1$ and $s = q+1$. Remark that this argument only depends on *r* being odd.

If $o \in O$ is one of these $q+1$ ovals, we see that the other *q* ovals intersect *O* in *q* distinct points, as no point is incident with more than two elements from $L \cup O$. This immediately implies that the unique line in *L* must be tangent to *o*. As *O* was arbitrary, we conclude that the support of the codeword consists of one line and $q+1$ ovals tangent to it. By Lemma 4.6 this indeed gives rise to a codeword, as every point not on the line is incident with zero or two ovals.

**Case II** ${\underline{q}}$ **even** The situation is slightly different. Since $q+2$ is even now, either *r* and *s* are both odd, or both even. When *r* is odd, we can reuse the argument from before to find the configuration of $q+1$ ovals tangent to a line. However, by Lemma 4.6 we know that these $q+1$ ovals are all incident with a unique point, which is hence covered $q+1$ times, a contradiction.

So suppose that *r* and *s* are even. Any line in *L* is intersected by the $r-1$ other lines in *L*, leaving $q+1-(r-1)$ points to be covered by the ovals in *O*, which is an even number. We see that we must have an even number of tangent ovals to this line. Similarly for a line not in *L*, we observe that it is intersected an even number of times by the *r* lines in *L* and hence it should have an even number of intersections with the ovals in *O*, leading again to an even number of tangent ovals. In summary, every line in ${{\,\mathrm{PG}\,}}(2,q)$ is incident with an even number of tangent ovals. Now consider any line $\ell$ and recall that the ovals tangent to $\ell$ are concurrent, say in the point $N_\ell$, by Lemma 4.6. However, as we saw before, $N_\ell$, like any other point, is covered zero or twice by the elements of $O \cup L$. It follows that $N_\ell$ is incident with zero or two ovals in *O* and hence that $\ell$ is tangent to zero or two ovals of *O*. So suppose that $s > 0$, meaning we have at least one oval in *O* and consider its $q+1$ tangent lines. Then each of these lines should have one more tangent oval, and all of these are distinct by Corollary 4.7, which means we find $s = q+2$ ovals forming a hyperoval of ovals. If $s = 0$, we find a dual hyperoval, concluding the theorem. $\square$

### Error-correction capability

It is well-known that the minimum distance of a code gives information about the decoding radius. This means that it reveals an upper bound on the amount of errors that can be always detected and corrected.

We would like to focus in this subsection here on the performance of the constructed MDPC code $C_2(\Uppi \sqcup \Upgamma )^\perp$ within one round of the bit-flipping decoding algorithm. We now adapt and apply Theorem 2.4 to the parity-check matrix *H* of $C_2(\Uppi \sqcup \Upgamma )^\perp$ given in (1).

#### Proposition 4.15

The maximum column intersection of the matrix *H* defined in (1) is $s_H=2$. Thus, after performing one round of the bit-flipping algorithm on *H* we can correct all the errors of weight at most $\lfloor \frac{q+1}{4}\rfloor$ in the code $C_2(\Uppi \sqcup \Upgamma )^\perp$.

#### Proof

From the construction of *H* we have that *H* consists of two matrices *A* and *B* which are the incidence matrices of points and lines and points and ovals of a projective bundle in ${{\,\mathrm{PG}\,}}(2,q)$, respectively. Clearly, both matrices *A* and *B* have a maximum column intersection equal to 1 as two distinct lines in a projective plane intersect in exactly one point and a similar property holds for every pair of distinct ovals of a projective bundle by definition. Since every line intersects an oval in at most 2 points, the maximum column intersection of the matrix *H* is at most 2. On the other hand, if we consider any two distinct points on an oval in the projective bundle, there always exists a line passing through them. Hence, $s_H=2$. The second part of the statement then follows directly from Theorem 2.4. $\square$

#### Remark 4.16

Observe that $s_H = 1$ for a parity-check matrix of size $(q^2+q+1)\times c$ and column weight $q+1$ implies $c \le q^2+q+1$. this can be seen by counting the tuples $\{(x,y,B) \,\, | \,\, x,y \in B\}$ in two ways. Thus, the value $s_H = 2$ is the best possible for $c > q^2+q+1$. Furthermore, compared to a random construction of MDPC code, our design guarantees a deterministic error-correction performance for one round of the bit-flipping decoding algorithm. In particular, for the random model proposed in [34] it was proved that the expected value of $s_H$ is ${\mathcal {O}}(\frac{\log n}{\log \log n})$. Hence, our construction guarantees an error-correction capability for one round of the bit-flipping algorithm which improves the random construction by a factor ${\mathcal {O}}(\frac{\log n}{\log \log n})$.

Additionally, we have implemented the parity-check matrix for our MDPC code design as well as the bit-flipping decoding algorithm. We were interested if we could correct even more errors than the number guaranteed in Proposition 4.15. Since the bit-flipping decoding algorithm is only dependent on the syndrome and not on the actual chosen codeword, we took the all-zero codeword and added a pseudo-random error-vector of a fixed weight $wt(e) \ge \lfloor \frac{q+1}{4} \rfloor$. We have generated $10^5$ distinct error vectors. Each of these error vectors then was used to run one round of the bit-flipping decoding algorithm for all the three different families of MDPC codes that we have constructed. It turned out that the codes constructed from each of the three types of projective bundles—circumscribed, inscribed, self-polar—showed exactly the same error-correction performance.

Finally, we have computed the probability of successful error-correction for the parameters $q \in \lbrace 5, 7, 9, 11, 13, 17, 19, 23, 25 \rbrace$. The following results were obtained for the different error weights.

**Table 1:** Probability to correctly decode a received word, with error-weight $\lfloor \frac{q+1}{4} \rfloor + i$ for $i=1, 2, 3$, after one round of the bit-flipping decoding algorithm

*q*	$\lfloor \frac{q+1}{4} \rfloor + 1$ errors	$\lfloor \frac{q+1}{4} \rfloor + 2$ errors	$\lfloor \frac{q+1}{4} \rfloor + 3$ errors
5	50.82%	0.16%	–
7	7.02%	0.34%	–
9	79.31 %	3.86%	–
11	43.83%	0.19%	–
13	90.4%	14.4%	–
17	97.2%	57.8%	7.8%
19	91.8%	42.6%	10.7%
23	97.86 %	77.66%	31.3%
25	99.87%	95.3%	71.25%

Table 1 shows that the probability to correct even more errors grows as we increase *q*. This is due to the fact, that for small *q* we reach the unique decoding radius much faster.

In the following we show some empirical results on the success probability after performing more than one round of the bit-flipping decoding algorithm (Tables 2, 3).

**Table 2:** Probability to correctly decode a received word, with error-weight $\lfloor \frac{q+1}{4} \rfloor + i$ for $i=1, 2, 3, 4$, after two rounds of the bit-flipping decoding algorithm

*q*	$\lfloor \frac{q+1}{4} \rfloor + 1$	$\lfloor \frac{q+1}{4} \rfloor + 2$	$\lfloor \frac{q+1}{4} \rfloor + 3$	$\lfloor \frac{q+1}{4} \rfloor + 4$
5	64.52%	3.45%	–	–
7	31.58%	4.75%	–	–
9	93.41 %	30.77%	–	–
11	43.83%	0.19%	–	–
13	99.45%	84.15%	35.52%	0.55%
17	100%	99.35%	84.69%	36.81%

**Table 3:** Probability to correctly decode a received word, with error-weight $\lfloor \frac{q+1}{4} \rfloor + i$ for $i=1, 2, 3, 4$, after three rounds of the bit-flipping decoding algorithm

*q*	$\lfloor \frac{q+1}{4} \rfloor + 1$	$\lfloor \frac{q+1}{4} \rfloor + 2$	$\lfloor \frac{q+1}{4} \rfloor + 3$	$\lfloor \frac{q+1}{4} \rfloor + 4$
5	64.52%	3.45%	–	–
7	45.61%	5.26%	–	–
9	94.51%	32.97%	–	–
11	93.23%	33.84%	8.27%	–
13	99.45%	94.54%	44.36%	1.64%
17	100%	100%	93.49%	50.81%

#### Remark 4.17

In [34] the author analyzed also the error-correction performance after two rounds of the bit-flipping decoding algorithm. More precisely he estimated the probability that one round of the algorithm corrects enough errors so that in the second round all remaining errors will be correctable. Following the notation of that paper, let us denote by *S* the number of errors left after one round of the bit-flipping algorithm. Assuming that we have an MDPC code of length *n* and of type (*v*, *w*), where both *v* and *w* are of order $\Theta (\sqrt{n})$, the probability that *S* is at least a certain value $t'$ satisfies the following inequality:

6 $$\begin{aligned} {\mathbb {P}}\left( S \ge t' \right) \le \frac{1}{\sqrt{t'}} \mathrm{e}^{\frac{t' v}{4} \ln (1 - \mathrm{e}^{-\frac{4wt}{n}}) + \frac{t'}{8}\ln (n) + O\left( t'\ln (t'/t) \right) }, \end{aligned}$$

where $t = \Theta (\sqrt{n})$ is the initial amount of errors that were introduced.

We have seen in Proposition 4.15, that performing one round of the bit-flipping algorithm to a parity-check matrix *H* of $C_2(\Uppi \sqcup \Upgamma )^\perp$ we can correct $\lfloor \frac{q+1}{4} \rfloor$ errors. Therefore, a second round of the bit-flipping is able to correct completely if after one round there are no more than $\lfloor \frac{q+1}{4} \rfloor$ errors left. Applying (6) for $t' = \lfloor \frac{q+1}{4} \rfloor$ to the parity-check matrix *H* of $C_2(\Uppi \sqcup \Upgamma )^\perp$ given in (1), we obtain that we can successfully correct every error of weight $t=\Theta (\sqrt{n})$ after two rounds of the bit-flipping decoding algorithm with probability $e^{-\Omega (n)}.$

## Generalizations

Since our aim is to have more flexibility in the parameters, here we generalize the approach of Sect. 4, by considering several disjoint projective bundles.

Let $t>1$ be a positive integer and let us fix a Desarguesian projective plane $\Uppi ={{\,\mathrm{PG}\,}}(2,q)$. Let $\Upgamma _1,\ldots , \Upgamma _{t}$ be disjoint^1^ projective bundles of conics in $\Uppi$. Since we want $s_H$ to be low, we cannot take projective bundles of ovals in general, as for example two ovals in ${{\,\mathrm{PG}\,}}(2,q)$, *q* even, could intersect in up to *q* points: take any oval, add the nucleus and delete another point to find a second oval intersecting it in *q* points. In Proposition 5.3 we will see that by choosing conics, we find $s_H = 4$. Furthermore, the number *t* of disjoint projective bundles cannot be chosen arbitrarily. However, the restrictions we have on *t* will not affect our intent to construct MDPC codes; see the discussion in Remark 5.4 for the admissible values of *t*.

Let us denote by *A* the incidence matrix of $\Uppi$ and by $B_i$ the incidence matrix of the projective bundle $\Upgamma _i$, for each $i\in \{1,\ldots ,t\}$. We then glue together all these matrices and consider the code $C_2(\Uppi \sqcup \Upgamma _1\sqcup \ldots \sqcup \Upgamma _t)^\perp$ to be the binary linear code whose parity-check matrix is

7 $$\begin{aligned} H_{q,t}:=\left( A \mid B_1 \mid \cdots \mid B_t \right) . \end{aligned}$$

As already discussed, it is important to specify which parity-check matrix of a code we consider when we study the decoding properties, since the bit-flipping algorithm depends on the choice of the parity-check matrix.

We focus now on the parameters on the constructed codes. We first start with a result on the dimension of the code $C_2(\Uppi \sqcup \Upgamma _1\sqcup \ldots \sqcup \Upgamma _t)^\perp$

### Proposition 5.1

Let $\Uppi ={{\,\mathrm{PG}\,}}(2,q)$ be a Desarguesian projective plane of order *q* and let $\Upgamma _1,\ldots ,\Upgamma _{t}$ be *t* projective bundles in $\Uppi$. Then,

$$\begin{aligned} \dim \left( C_2(\Uppi \sqcup \Upgamma _1\sqcup \ldots \sqcup \Upgamma _t)^\perp \right)&= t(q^2+q+1)+1&\text{ if } q \text{ is } \text{ odd, } \\ \dim \left( C_2(\Uppi \sqcup \Upgamma _1\sqcup \ldots \sqcup \Upgamma _t)^\perp \right)&\ge (t+1)(2^{2h}-3^h+2^h)+t&\text{ if } q=2^h. \end{aligned}$$

### Proof

The proof goes as for Proposition 4.13.

${{{\underline{\textit{\textbf{Case I}}}}}}\,{\underline{q}}\,{{{\underline{\varvec{odd}}}}}$ We know from Theorem 3.4 that ${{\,\mathrm{rk}\,}}(A) = q^2+q$, which gives us the lower bound ${{\,\mathrm{rk}\,}}(H_{q,t}) \ge {{\,\mathrm{rk}\,}}(A) = q^2+q$.

On the other hand, since *A* and each matrix $B_i$ have the all-one vector in its left-kernel, we have that also $H_{q,t}$ has a nontrivial left-kernel, and hence ${{\,\mathrm{rk}\,}}(H_{q,t})=q^2+q$, yielding

$$\begin{aligned} \dim \left( C_2(\Uppi \sqcup \Upgamma _1\sqcup \ldots \sqcup \Upgamma _t)^\perp \right) =(t+1)(q^2+q+1)-{{\,\mathrm{rk}\,}}(H_{q,t})= t(q^2+q+1)+1. \end{aligned}$$

**Case II** ${\underline{q}}$ **even** Let us write $q=2^h$. In this case, we have that the all-one vector belongs to the column spaces of each matrix $B_i$. Therefore,

$$\begin{aligned} {{\,\mathrm{rk}\,}}(H_{q,t})\le {{\,\mathrm{rk}\,}}(A)+\sum _{i=1}^t{{\,\mathrm{rk}\,}}(B_i)-t. \end{aligned}$$

Thus, we obtain

$$\begin{aligned} \dim \left( C_2(\Uppi \sqcup \Upgamma _1\sqcup \ldots \sqcup \Upgamma _t)^\perp \right)&=(t+1)(q^2+q+1)-{{\,\mathrm{rk}\,}}(H_{q,t})\\&\ge (t+1)(q^2+q+1)-{{\,\mathrm{rk}\,}}(A)-\sum _{i=1}^t{{\,\mathrm{rk}\,}}(B_i)+t \\&=(t+1)\dim (C_2(\Uppi )^\perp )+t\\&=(t+1)(2^{2h}-3^h+2^h)+t , \end{aligned}$$

where the last equality comes from Theorem 3.4. $\square$

Also in this general case we can study the minimum distance of the code $C_2(\Uppi \sqcup \Upgamma _1\sqcup \ldots \sqcup \Upgamma _t)^\perp$, generalizing the result on the minimum distance obtained when $t=1$ in Theorem 4.14. However, this time we are only able to give a lower bound.

### Proposition 5.2

The minimum distance of $C_2(\Uppi \sqcup \Upgamma _1\sqcup \ldots \sqcup \Upgamma _t)^\perp$ is at least $\big \lceil \frac{q+2}{2}\big \rceil$.

### Proof

The proof goes in a similar way as the one of Theorem 4.14. Take a codeword of minimum weight in $C_2(\Uppi \sqcup \Upgamma _1\sqcup \ldots \sqcup \Upgamma _t)^\perp$ and consider its support. This is a set *L* of *r* lines and a set $O_i$ of $s_i$ ovals for each $i \in \{1,\ldots ,t\}$ such that every point in ${{\,\mathrm{PG}\,}}(2,q)$ is incident with an even number of these elements. We will show that $r+s_1+\ldots +s_t \ge \frac{q+2}{2}$.

Let $a_i$, $0 \le i \le (t+1)(q+1)$, be the number of points that are covered *i* times, then we can double count the tuples $(P), (P,E_1), (P,E_1,E_2)$, where *P* is a point and $E_1,E_2 \in L \cup O_1\cup \ldots \cup O_t$ are lines or ovals incident with this point. Remark that by assumption $a_i = 0$ whenever *i* is odd. We find the following three expressions:

8 $$\begin{aligned} \sum _{i=0}^{(t+1)(q+1)} \!\!\!a_i&= q^2+q+1 \end{aligned}$$

9 $$\begin{aligned} \sum _{i=0}^{(t+1)(q+1)} \!\!\!ia_i&= \bigg (r+\sum _{i=1}^ts_i\bigg )(q+1) \end{aligned}$$

10 $$\begin{aligned} \sum _{i=0}^{(t+1)(q+1)} \!\!\!i(i-1)a_i&\le r(r-1)+\sum _{i=1}^ts_i(s_i-1)+2r\bigg (\sum _{i=1}^ts_i\bigg )+4\bigg (\sum _{1\le i<j\le t}s_is_j\bigg ), \end{aligned}$$

as two conics intersect in at most 4 points by Bézout’s theorem. Subtracting (9) from (10) we obtain

$$\begin{aligned} 0 \le \!\!\sum _{i=0}^{(t+1)(q+1)} \!\!\! i(i-2)a_i = \bigg (r+\sum _{i=1}^ts_i\bigg )\bigg (r-q-2+\sum _{i=1}^ts_i\bigg )+2\bigg (\sum _{1\le i<j\le t}s_is_j\bigg ). \end{aligned}$$

One can easily check that this last quantity is in turn at most

$$\begin{aligned} \bigg (r+\sum _{i=1}^ts_i\bigg )\bigg (2r-q-2+2\sum _{i=1}^ts_i\bigg ), \end{aligned}$$

which then implies

$$\begin{aligned} r+\sum _{i=1}^ts_i \ge \frac{q+2}{2}. \end{aligned}$$

$$\square $$

As a direct consequence of Proposition 5.2 we have that in principle it should be possible to correct at least $\big \lfloor \frac{q}{4} \big \rfloor$ errors in the code $C_2(\Uppi \sqcup \Upgamma _1\sqcup \ldots \sqcup \Upgamma _t)^\perp$ when *q* is even, and at least $\big \lfloor \frac{q+1}{4}\big \rfloor$ when *q* is odd. However, also in this case, when running one round of the bit-flipping algorithm on the matrix $H_{q,t}$ given in (7), we only correct a smaller fraction of them, as the following result shows.

### Proposition 5.3

The maximum column intersection of the matrix $H_{q,t}$ defined in (7) is at most 4. Thus, after performing one round of the bit-flipping algorithm on $H_{q,t}$ we can correct all the errors of weight at most $\lfloor \frac{q+1}{8}\rfloor$ in the code $C_2(\Uppi \sqcup \Upgamma _1\sqcup \ldots \sqcup \Upgamma _t)^\perp$.

### Proof

The maximum column intersection is given by the maximum number of points lying in the intersection of elements in $\Uppi \sqcup \Upgamma _1\sqcup \ldots \sqcup \Upgamma _t$. Each pair of lines intersects in exactly a point, and the same holds for every pair of conics belonging to the same projective bundle, since each projective bundle is itself (ismorphic to) a projective plane. Moreover, every line intersects a conic in at most two points, and we have already seen that each pair of conics meets in at most 4 points. Hence, the maximum column intersection of $H_{q,t}$ is at most 4. The second part of the statement directly follows from Theorem 2.4. $\square$

### Remark 5.4

At this point it is natural to ask whether it is possible to construct disjoint projective bundles, and—if so—how many of them we can have. It is shown in [4, Theorem 2.2] that one can always find $(q-1)$ disjoint projective bundles when *q* is even, and $\frac{q^2(q-1)}{2}$ of them when *q* is odd. We want to remark that this is not a restriction, since we still want that our codes $C_2(\Uppi \sqcup \Upgamma _1\sqcup \ldots \sqcup \Upgamma _t)^\perp$ (together with the parity-check matrices $H_{q,t}$ of the form (7)) give rise to a family of MDPC codes. Thus, we are typically interested in family of codes where *t* is a constant and does not grow with *q*.

### Remark 5.5

This construction provides a better performance of (one round of) the bit-flipping algorithm compared to the one run on random constructions of MDPC codes explained in [34]. As already explained in Remark 4.16, the random construction of MDPC codes provides in average MDPC codes whose maximum column intersection is ${\mathcal {O}}(\frac{\log n}{\log \log n})$, and thus one round of bit-flipping algorithm corrects errors of weight at most ${\mathcal {O}}(\frac{\sqrt{n}\log \log n}{\log n})$ in these random codes. Hence, also the generalized constructions of codes from projective bundles have asymptotically better performance in terms of the bit-flipping algorithm.

## Further variations and comparisons

In the previous section we have shown that codes constructed using projective bundles have a very interesting combinatorial and geometric structure, which allows to determine the parameters and correction capability properties. In particular, we were able not only to determine the minimum distance of codes obtained using only a projective bundle, but we also classified their minimum weight codewords.

In terms of parameters, it is clear that there exist better families of linear codes. Indeed, the codes presented in Sect. 4 have minimum distance $\mathcal O(\sqrt{n})$ and hence they are certainly not asymptotically good. Nevertheless, in addition to the geometric structure, the combinatorial characterization also allows a very efficient storage.

In particular, let $q=2^h$ be even, and consider a projective plane $\Uppi \cong {{\,\mathrm{PG}\,}}(2,2^h)$ and the inscribed projective bundle $\Upgamma$ in ${{\,\mathrm{PG}\,}}(2,q)$. Then, it is readily seen that $B=A^2$. When instead one has that $\Upgamma$ is a circumscribed bundle, then $A=B^2$. In both cases, we have that the code $C_2(\Uppi \sqcup \Upgamma )^\perp$ is (equivalent to) a code with parity-check matrix of the form

$$\begin{aligned} H=(\,A \,|\, A^2\, ). \end{aligned}$$

Since we have seen in the proof of Proposition 4.13 that for *q* even we have ${{\,\mathrm{rk}\,}}(H)=2{{\,\mathrm{rk}\,}}(A)-1$, then one can verify that the code $C_2(\Uppi \sqcup \Upgamma )^\perp$ is made by two copies of $C_2(\Uppi )^\perp$, together with the all-one vector. Formally, we have

$$\begin{aligned} C_2(\Uppi \sqcup \Upgamma )^\perp =\left\{ (c+\lambda x \mid c'+\lambda x) \,|\,c,c' \in C_2(\Uppi )^\perp , \lambda \in {\mathbb {F}}_2\right\} , \end{aligned}$$

where *x* denotes the all-one vector of length $(2^{2h}+2^h+1)$. Thus, for coding theoretic purposes, this code is not more interesting than just the code $C_2(\Uppi )^\perp$ itself. However, we remark that we included nevertheless the results also when *q* is even, in order to have a complete study and for their intrinsic geometrical interest.

### Another code construction

Now, for the case of *q* odd, we present another construction that only deals with the incidence matrix *A* of a projective plane $\Uppi \cong {{\,\mathrm{PG}\,}}(2,q)$. We define the code $D_p(\Uppi )^\perp$ to be the code over ${\mathbb {F}}_p$ whose parity-check matrix is

11 $$\begin{aligned} H:=\left( \begin{array}{c|c} I &{} \,A \, \\ \hline A^{\top } \!&{} \, I \end{array}\right) .\end{aligned}$$

For the rest of this section, we will always use *H* to denote the matrix in (11). The following lemma is a straightforward computation using Gaussian elimination, whose proof is left to the reader.

#### Lemma 6.1

Let *q* be an odd prime power and let $\Uppi$ be a projective plane of order *q*. The code $D_p(\Uppi )^\perp$ coincides with the right kernel of

$$\begin{aligned} \left( \begin{array}{c|c} \, I\,\, &{} A \\ \hline \,0\,\, &{} J +(q+1)I\end{array} \right) . \end{aligned}$$

In particular, if *q* is odd and $p=2$ the following matrices are parity-check matrices for the code $D_2(\Uppi )^\perp$:

$$\begin{aligned} H_1:=\left( \begin{array}{c|c} \, I\,\, &{} \,A \,\\ \hline \,0\,\, &{} \,x\, \end{array} \right) , \qquad H_2:=\left( \begin{array}{c|c} \, A^\top \, &{} \,\,I\, \\ \hline \,x\, &{} \,\,0\,\end{array} \right) , \end{aligned}$$

where *x* is the all-one vector of length $q^2+q+1$.

Observe that the columns of the matrix *H* in (11), as well as those of $H_1$ and $H_2$ of Lemma 6.1, can be thought as corresponding to lines and points of $\Uppi$. In particular, the first $q^2+q+1$ columns of *H* (resp. $H_1$ and $H_2$) correspond to the points of $\Uppi$ and the last $q^2+q+1$ columns of *H* (resp. $H_1$ and $H_2$) correspond to the lines of $\Uppi$. Furthermore, as we did in Theorem 4.14, since we are only considering binary codes, we also identify the supports of the codewords with the corresponding sets of lines and/or points. By the definition of its parity-check matrix, it follows that a codeword in $D_2(\Uppi )^\perp$ corresponds to a set of points and lines such that every point and line in ${{\,\mathrm{PG}\,}}(2,q)$ is incident with an even number of elements, where a point (resp. line) is incident with itself but no other point (resp. line). Furthermore, by Lemma 6.1, we see from considering the last row that both the number of points and the number of lines in a subset corresponding to a codeword should be even.

#### Theorem 6.2

Let *q* be an odd prime power, and let $\Uppi$ be a projective plane of order *q*. Then, $D_2(\Uppi )^\perp$ is a $[2(q^2+q+1), q^2+q,2q+2]_2$ code. Furthermore, the codewords of minimum weight correspond to one of the following cases:

- Any two distinct lines $\ell _1$ and $\ell _2$ of $\Uppi$ and the set of points on one of $\ell _1$ or $\ell _2$ but not both;
- Dually, any two distinct points $P_1, P_2$ and the set of lines through one of $P_1$ or $P_2$ but not both;
- An oval and its $q+1$ tangent lines.

#### Proof

The fact that $\dim (D_2(\Uppi )^\perp ) =q^2+q$ directly follows from Lemma 6.1, since it is immediate to see that the matrices $H_1$ and $H_2$ have full row rank equal to $q^2+q+2$.

Thus, we only need to determine the minimum distance *d* of $D_2(\Uppi )^\perp$. The three families of subsets mentioned above indeed define codewords of $D_2(\Uppi )^\perp$, which can be seen by the discussion preceding the theorem. Therefore, the minimum distance is at most the weight of any of these three, which is $2q+2$ for all of them. We will show that this is the minimum possible and characterize the three types as the only codewords of this weight.

So take a set of *r* points and *s* lines and assume $r + s \le 2q+2$. We cannot have $r = 0$ or $s = 0$ as can be seen geometrically or by Lemma 6.1, so by their evenness we find $r,s \ge 2$. Since any line contains $q+1$ points and any two lines intersect in one point, we see that there are at least $s(q+1)-s(s-1)$ points which are covered an odd number of times. This means that $r \ge s(q+2-s)$, and hence $r+s \ge s(q+3-s)$. The minimum is attained for $s\in \{2,q+1\}$ and equals $2q+2$ so the minimum distance is indeed $2q+2$.

We now characterize the codewords of minimum weight. When $s=2$, we have two lines $\ell _1,\ell _2$ and we need to add the 2*q* points on $(\ell _1\cup \ell _2)\setminus (\ell _1\cap \ell _2)$ as they are incident with an odd number of lines. This already adds up to $2q+2$ and this set indeed defines a codeword. Dually, when $r = 2$ the same argument shows that one finds the second type of minimum weight codeword. Finally when $r = s = q+1$ we find equality in the argument above which implies that there are no three lines concurrent, and dually that no three points are collinear. In other words, we find the union of an oval and a dual oval, which can only define a codeword if the dual oval consists exactly of the tangent lines of the oval, which gives the last type. $\square$

#### Corollary 6.3

The number of minimum weight codewords in $D_2(\Uppi )^\perp$ is equal to $q(q^2+q+1)(q^2+1)$.

#### Proof

By Theorem 6.2, the codewords of minimum weight are of three types. The number of codewords of the first type equals the number of pair of lines, and by duality is also equal to the number of codewords of the second type. They clearly sum up to $(q^2+q+1)q(q+1)$. The number of codewords of the last type coincides with the number of ovals in ${{\,\mathrm{PG}\,}}(2,q)$, which by Segre’s theorem [32] is equal to $q^2(q^3-1)$, i.e. the number of nondegenerate conics. This gives the desired result. $\square$

### Error-correction capability and experimental results

In the following result we derive the error-correction capability of (one round of) the bit-flipping algorithm.

#### Proposition 6.4

The intersection number of the matrix *H* defined in (11) is $s_H=2$. Thus, after performing one round of the bit-flipping algorithm on *H* we can correct all the errors of weight at most $\lfloor \frac{q+1}{4}\rfloor$ in the code $D_2(\Uppi )^\perp$.

#### Proof

It is clear that the intersection number is $s_H=2$. Hence, by Theorem 2.4, after one round of the bit-flipping algorithm on *H* we can correct all the errors of weight $\lfloor \frac{q+2}{4}\rfloor$, which is equal to $\lfloor \frac{q+1}{4}\rfloor$ since *q* is odd.$\square$

Thus, we have that the correction capability of one round of the bit-flipping algorithm for *H* as in (11) is the same as the one for *H* in (1). Hence, with these two choices of parity-check matrices and using one round of the bit-flipping algorithm, the codes $C_2(\Uppi \sqcup \Upgamma )^\perp$ and $D_2(\Uppi )^\perp$ have the same error-correction capability, at least for what concerning *all* the error patterns.

Nevertheless, similar to the experiments seen in Sect. 4.3 we were curious on how many additional errors we can add to the number of errors found in Proposition 6.4 and still be able to correct after one and more rounds of the bit-flipping decoding algorithm. The simulations were run under the exact same circumstances as for the code $C_2(\Uppi \sqcup \Upgamma )^\perp$ in order to be able to compare the results. The following tables show the success rate of correcting more than $\lfloor (q+1) / 4 \rfloor$ errors within up to four rounds of bit-flipping.

**Table 4:** Probability to correctly decode a received word, with error-weight $\lfloor \frac{q+1}{4} \rfloor + i$ for $i=1, 2, 3, 4$, after one round of the bit-flipping decoding algorithm

*q*	$\lfloor \frac{q+1}{4} \rfloor + 1$	$\lfloor \frac{q+1}{4} \rfloor + 2$	$\lfloor \frac{q+1}{4} \rfloor + 3$	$\lfloor \frac{q+1}{4} \rfloor + 4$
5	100%	51.61%	9.68%	1.61%
7	100%	64.91%	13.16%	6.14%
9	100%	100%	74.73%	42.86%
11	100%	100%	86.84%	53.01%
13	100%	100%	100%	92.08%
17	100%	100%	100%	100%

Table 4 already shows us that the code $D_2(\Uppi )^\perp$ based on a parity-check defined in (11) has a probability of 1 to correct one error more than the expected number of errors from Proposition 6.4 for every value of *q* presented in Table 4. If we increase the number of rounds of the bit-flipping algorithm we expect that more and more errors are correctable. This expectation is motivated by Theorem 6.2, stating that the minimum distance of $D_2(\Uppi )^\perp$ is given by $d(D_2(\Uppi )^\perp ) = 2q + 2$. Hence the unique decoding radius is $q+1$, which is about four times as large as the number of errors correctable within one round of bit-flipping. The Tables 5, 6 and 7 support this expectation.

**Table 5:** Probability to correctly decode a received word, with error-weight $\lfloor \frac{q+1}{4} \rfloor + i$ for $i=1, 2, 3, 4$, after two rounds of the bit-flipping decoding algorithm

*q*	$\lfloor \frac{q+1}{4} \rfloor + 1$	$\lfloor \frac{q+1}{4} \rfloor + 2$	$\lfloor \frac{q+1}{4} \rfloor + 3$	$\lfloor \frac{q+1}{4} \rfloor + 4$
5	100%	53.23%	51.61%	9.68%
7	100%	64.91%	63.16%	52.63%
9	100%	100%	84.62%	68.68%
11	100%	100%	99.25%	84.21%
13	100%	100%	100%	99.73%
17	100%	100%	100%	100%

**Table 6:** Probability to correctly decode a received word, with error-weight $\lfloor \frac{q+1}{4} \rfloor + i$ for $i=1, 2, 3, 4$, after three rounds of the bit-flipping decoding algorithm

*q*	$\lfloor \frac{q+1}{4} \rfloor + 1$	$\lfloor \frac{q+1}{4} \rfloor + 2$	$\lfloor \frac{q+1}{4} \rfloor + 3$	$\lfloor \frac{q+1}{4} \rfloor + 4$
5	100%	100%	90.32%	32.26%
7	100%	100%	76.32%	57.02%
9	100%	100%	100%	80.22%
11	100%	100%	100%	84.21%
13	100%	100%	100%	100%
17	100%	100%	100%	100%

**Table 7:** Probability for $D_2(\Uppi )^\perp$ to correctly decode a received word, with error-weight $\lfloor \frac{q+1}{4} \rfloor + i$ for $i=1, 2, 3, 4$, after four rounds of the bit-flipping decoding algorithm

*q*	$\lfloor \frac{q+1}{4} \rfloor + 1$	$\lfloor \frac{q+1}{4} \rfloor + 2$	$\lfloor \frac{q+1}{4} \rfloor + 3$	$\lfloor \frac{q+1}{4} \rfloor + 4$
5	100%	100%	98.38%	43.55%
7	100%	100%	83.33%	57.89%
9	100%	100%	100%	82.42%
11	100%	100%	100%	84.59%
13	100%	100%	100%	100%
17	100%	100%	100%	100%

### Comparisons

In this final section let us compare the two code constructions $C_2(\Uppi \sqcup \Upgamma )^\perp$ and $D_2(\Uppi )^\perp$ of MDPC codes. First of all, with both the constructions we are able to give deterministic results on the error-correction performance for one round of the bit-flipping decoder, which for the random construction in [34] is not possible.

The two codes $C_2(\Uppi \sqcup \Upgamma )^\perp$ and $D_2(\Uppi )^\perp$ have the same length and almost the same dimension. From Theorem 4.14 and Theorem 6.2, we know that the minimum distance of $D_2(\Uppi )^\perp$ is almost twice of the minimum distance of $C_2(\Uppi \sqcup \Upgamma )^\perp$. Hence $D_2(\Uppi )^\perp$, in general, is able to correct almost twice as many errors as $C_2(\Uppi \sqcup \Upgamma )^\perp$. Nevertheless, applying Theorem 2.4 to both of them, yields the same result for one round of the bit-flipping decoding algorithm. Furthermore, we observe that the number of nonzero entries of the parity-check matrix (1) for $C_2(\Uppi \sqcup \Gamma )^\perp$ is almost the same as the number of nonzero entries of the parity-check matrix (11) for $D_2(\Uppi )^\perp$. Since the complexity of the bit-flipping decoder is depending on the length *n*, the maximal number of iterations and the number of nonzero entries, we deduce that it will have roughly the same run time.

To conclude we can say, that the construction using projective bundles shows interesting properties from a mathematical viewpoint. Nevertheless, from a coding theoretic perspective the code $D_2(\Uppi )^\perp$, which has the same length and almost the same dimension as $C_2(\Uppi \sqcup \Upgamma )^\perp$, shows a higher error-correction performance with respect to the bit-flipping decoder. In fact, its minimum distance is almost twice as large and hence the unique decoding radius is larger.

## Conclusion

In this paper we proposed a new construction of a family of moderate-density parity-check codes arising from geometric objects. Starting from a Desarguesian projective plane $\Uppi$ of order *q* and a projective bundle $\Upgamma$ in $\Uppi$, we constructed a binary linear code whose parity-check matrix is obtained by concatenating the incidence matrices of $\Uppi$ and $\Upgamma$. We observed that we can construct these two matrices taking the circular shifts of two perfect difference sets modulo $(q^2+q+1)$, providing a natural structure as a quasi-cyclic code of index 2. Hence, the storage complexity is linear in the length and the encoding can be achieved in linear time using linear feedback shift registers. Furthermore, the underlying geometry of $\Upgamma$ and $\Uppi$ allowed us to study the metric properties of the corresponding code, and we could determine its exact dimension and minimum distance, as well as its minimum weight codewords. We then analyzed the performance of the bit-flipping algorithm showing that it outperforms asymptotically the one of the random construction of codes obtained in [34]. We then generalized the construction of this family of codes by concatenating the incidence matrices of several disjoint projective bundles living in the Desarguesian projective plane $\Uppi$. In this case we were able to provide lower bounds on the parameters of the obtained codes exploiting their geometric properties. Nevertheless, we could still show that one round of the bit-flipping algorithm has the best asymptotic performance in terms of error-correction capability for the given parameters of the defining parity-check matrix. Finally, we gave an alternative construction of binary codes whose parity check matrices only use the incidence matrix of $\Uppi$ and its transpose. We determined the parameters of these codes and characterized the minimum weight codewords. The error-correction performance was then empirically studied by implementing the parity-check matrix and running the bit-flipping decoding for several iterations. The empirical results showed that this alternative construction outperforms the construction using projective bundles in terms of being able to successfully decode more errors. Hence, adding redundant rows to the parity-check matrix might seem to be a promising tool to improve the error-correction performance. Future research, thus, might study the case, when adding additional linearly dependent rows to the parity-check matrix of $C_2(\Uppi \sqcup \Upgamma )^\perp$ and analyzing its performance for the bit-flipping decoder. In particular, it would be very interesting to understand whether there is a systematic way to add redundant parity-check equations in order to maximize the bit-flipping decoder performance.

## Footnotes

1 Here with “disjoint” we mean that any two distinct projective bundles have no common oval.
